# Skin Autofluorescence in Systemic Sclerosis Is Related to the Disease and Vascular Damage: A Cross-Sectional Analytic Study of Comparative Groups

**DOI:** 10.1155/2015/837470

**Published:** 2015-12-31

**Authors:** Jolanta Dadoniene, Alma Cypiene, Ligita Ryliskyte, Rita Rugiene, Kristina Ryliškiene, Aleksandras Laucevičius

**Affiliations:** ^1^Vilnius University Rheumatology Center, Santariskiu 2, Vilnius, Lithuania; ^2^State Research Institute for Innovative Medicine, Zygimantu 9, Vilnius, Lithuania; ^3^Vilnius University Center of Cardiology and Angiology, Santariskiu 2, Vilnius, Lithuania; ^4^Vilnius University Center of Neurology, Santariskiu 2, Vilnius, Lithuania

## Abstract

Skin autofluorescence (AF), a relatively simple and time saving procedure, measures the accumulation of advanced glycation end (AGE) products. The importance in autoimmune rheumatic diseases, particularly, systemic sclerosis (SSc), has not been evaluated yet. The aim of our study was to examine the skin AF in the context of SSc patients and to analyse the relations between skin AF and other surrogate measures of atherosclerosis. Forty-seven patients with SSc and 47 healthy volunteers were included in this study as controls. Patients and controls underwent common carotid artery wall assessment, arterial stiffness and wave reflection measurements, laser Doppler measurements of capillary flow, assessment of endothelial function by brachial ultrasound, peripheral arterial tonometry, and AGE measurement by skin AF. Wall properties of the common carotid arteries and wave reflection measurements were not affected in these study patients compared to controls while measures reflecting small capillary flow were altered. The accumulation of AGE products measured by skin AF was more prominent in SSc patients than in healthy controls. AGE products' score was significantly associated with carotid radial pulse wave velocity, intima media/carotid artery diameter ratio, capillary flow percentage change during occlusion, and the disease itself in a multivariate linear analysis model.

## 1. Background

There is an increasing and proved evidence of autoimmune rheumatic diseases being associated with early atherosclerosis and cardiovascular events occurring more often if compared to the same strata from a population [1, 2]. Autoantibodies, autoantigens, proinflammatory cytokines, and infectious agents play a role in that process [3, 4]. Involvement of autoimmunity in the pathogenesis of accelerated atherosclerosis in rheumatic diseases results in early changes of vascular endothelium but the possibilities to reveal them at preventable stage are few. There have been significant advances in the noninvasive assessment of endothelial function, atherosclerosis, and vascular stiffness in rheumatic diseases by determining flow-mediated dilatation, intima media thickness of carotids, and pulse wave velocity although none of them proved to be a trustworthy prognostic marker and some of them are hardly applicable in practice [2]. Skin autofluorescence, relatively simple and time saving procedure, is related to the accumulation of AGE products and is one of the strongest markers to predict cardiovascular events in diabetes, renal insufficiency, and atherosclerosis itself [5]. Skin AF was investigated in few rheumatic diseases, lupus erythematosus, and rheumatoid arthritis, in particular. Its importance in SSc has not been acknowledged yet, and it evolved with two conflicting publications by Hettema et al. in 2011 [6] and Murray et al. in 2012 [7]. The aim of our study is to examine the skin AF in the context of SSc patients and to analyse the relations between skin AF and other surrogate measures of atherosclerosis.

## 2. Methods

Forty-seven SSc patients who met the American College of Rheumatology criteria for the disease were included in this cross-sectional study. Forty-seven age (± 3 years) and gender matched healthy volunteers were included into this study as control subjects. The serum levels of total cholesterol (TC), triglycerides, high density lipoprotein cholesterol (HDL-C), and low density lipoprotein cholesterol (LDL-C) were assessed in all patients and healthy controls. Patients with a history of cardiovascular event, hypertension, smokers, or patients suffering from conditions that affect the lipid profile, such as diabetes mellitus, hypothyroidism, liver or kidney diseases, and obesity, were not included. Patients continued to receive medication for the disease treatment.

All patients and healthy controls underwent detailed assessment of arterial function and wall properties, which consisted of carotid wall assessment, arterial stiffness and pressure wave reflection measurements, laser Doppler measurements of capillary flow, and endothelial function assessment by brachial ultrasound and peripheral arterial tonometry. Vascular assessment was carried out in supine position after 20 minutes bed rest in a temperature-controlled room at 23°C. Study subjects abstained from drinking alcoholic beverages or coffee and from taking vasoactive drugs for 12 hours before the examination. Measurements of autofluorescence were performed on the inner side of the lower arm below the elbow on a single measurement site.

### 2.1. Common Carotid Artery Wall Assessment

Common carotid intima media thickness (IMT), cross-sectional carotid artery distensibility (both calculated in *μ*m), and nondimensional index Quality Carotid Stiffness of common carotid artery (CCA) were measured using high-resolution echo-tracking technology (Art. Lab, Esaote Europe B.V., Maastricht, Netherlands) at 1 cm proximal to the carotid bifurcation along 4 cm arterial segment. Ratio between IMT and diameter of CCA was calculated and mean values of right and left side CCA measurements were used in the analysis.

### 2.2. Arterial Stiffness and Wave Reflection Measurements

Parameters of arterial stiffness and wave reflection were assessed by applanation tonometry (SphygmoCor, AtCor Medical, version 8.0, Sydney, Australia). Radial, carotid, and femoral pressure waveforms were recorded for 20 seconds each with single transducer synchronized with ECG R wave, after obtaining high quality waveform. The distance between the arterial sites was measured on the body using a tape measure. Carotid femoral pulse wave velocity (cfPWV), the “gold standard” parameter of aortic stiffness, and carotid radial pulse wave velocity (crPWV) representing arterial stiffness in peripheral arteries were calculated automatically as the distance divided by time (meters per second) and multiplied by 0.8 for cfPWV according to European expert consensus [8]. Aortic pressure waveform with calculation of heart-rate adjusted aortic augmentation index (AIx) was automatically derived from radial pressure waveform using previously validated transfer function.

### 2.3. Laser Doppler Measurements of Capillary Flow

Cutaneous capillary blood flow was measured using a laser Doppler (LD) flowmeter (PeriFlux System 5000, Perimed, Järfälla, Sweden). A laser probe (PR457) was attached to the dorsal surface of the distal pad of the third left finger and maintained in the same position during all LD measurements. Data from LD flowmeter were interfaced to a personal computer through a converter using Perisoft data acquisition software. LD blood flow measurements were recorded in mV, which are directly related to blood flow in the microcirculation of the surface tissue. LDM were undertaken in the following sequence: postocclusive hyperaemia with a 20-minute recovery period, thermal hyperaemia with a 20-minute recovery period, and cold stimulation. Blood pressure and heart-rate measurements were performed before every subsequent phase of study.

#### 2.3.1. Postocclusive Hyperaemia

After 10 minutes of rest, to allow for the measurement of baseline cutaneous capillary perfusion, digital blood flow was occluded for 5 minutes by inflating a cuff placed on the left arm to 100 mmHg above the systolic blood pressure. The cuff was then released and the flow responses were recorded. The amplitude of the response was determined by the peak hyperemic perfusion, expressed as an absolute value of perfusion units (PU). The kinetics of the response was described by the time to peak hyperaemia, expressed in seconds, and percent change in capillary flow.

#### 2.3.2. Thermal Response

The microvascular response to local heating was measured 20 minutes later. The baseline flow was measured by PR457 laser probe for 5 minutes. Then, the probe was heated to 44°C for 30 minutes. The amplitude of the response was expressed as perfusion units (PU). The rest flow before heating, maximal flow in response to heating, and time to maximal flow were assessed from the capillary flow curves recorded [9].

#### 2.3.3. Cold Stimulation

After 20 minutes, cold stimulation was performed. The baseline flow was measured by PR457 laser probe for 5 minutes. Then, left hand was immersed in 12°C water. Following 5 minutes of cooling, hand was pulled out of the water and dried and the probe was heated to 32°C for 5 minutes. The rest flow before cooling, minimal perfusion in response to cooling, and time to minimal perfusion were assessed.

### 2.4. Assessment of Endothelial Function by Brachial Ultrasound and Peripheral Arterial Tonometry

#### 2.4.1. Ultrasound Assessment of Endothelial Function in Brachial Artery

The endothelium-dependent flow-mediated dilatation (FMD) test in a brachial artery was performed by using ultrasound system with high-resolution 12 MHz linear-array transducer and echo-tracking mode (Prosound *α*-10, Aloka, Japan). The brachial artery diameter was measured at rest and after 5 minutes of ischemia introduced by inflating pneumatic tourniquet placed on the forearm by 100 mmHg above the systolic blood pressure. Endothelium-dependent vasodilatation was semiautomatically calculated as a percent change in arterial diameter at the baseline and during postocclusive reactive hyperaemia.

#### 2.4.2. Peripheral Arterial Tonometry

Endothelial function in microcirculation was assessed by peripheral arterial tonometry (Endo-PAT 2000 system, Itamar Medical, Israel) [10]. The Endo-PAT probes that impart a uniform pressure field to the distal two-thirds of the finger were placed on the index finger of each arm. While nondominant arm was tested, the contralateral arm provided data on the systemic vascular changes, such as changes in sympathetic tone. Endothelium-dependent postocclusive reactive hyperaemia in the tested arm was induced by 5-minute ischemic test as described above. The reactive hyperaemia index (RHI) was calculated as log ratio of the pulse amplitude augmentation between the tested and control arm.

### 2.5. Skin Autofluorescence

Skin AF for measuring accumulation of AGEs was assessed by AF reader (AGE Reader; Diagnoptics, Groningen, Netherlands) as described in user's manual of this device and detailed in related publication [11]. During the subject measurement, the actual measurement with ultraviolet-A light was performed. After warming up and before the start of the measurement, a window appears on AGE Readers hand supporting device. Measurements were taken on the dominant arm avoiding traumatic skin damage. The arm was tightly placed in a device and hereby fully covered the window of the light. The measurement usually takes 20–30 seconds. For increased accuracy, we used triple measurement option that can be selected from the settings of the AGE Reader meaning three measures in a row of the same site. The AF values were calculated from the measurements on the patient by the software. AF was calculated as the ratio of the light intensity in the 420–600 nm wavelength range and the light intensity in the 300–420 nm wavelengths. The reflection factor is the ratio of mean intensities that are measured at the skin divided by the ratio that would occur on an external white standard. Its value is a weighted reflection around the mean weighted wavelength (~370 nm). AF values should always account for age as it is well known that age is the most important factor determining AGE content in different organs. As presented by AGE Reader developers, AF measurement for healthy group within age category of 40–49 years and 50–59 years is set to be 1.8 (0.4) and 2.1 (0.3), in respect.

### 2.6. Statistical Analysis

Quantitative variables if normally distributed were expressed as means (standard deviation (SD)) and Student's *t*-test was used for comparisons between groups. If the distribution was abnormal, the median and range were presented and Mann-Whitney test was used. The qualitative variables are expressed as numbers and percentages. To assess the influence of age and tested measurements on the outcome variable skin autofluorescence, the stepwise multiple regression analysis was performed. Statistical significance was set up at level below 5% for univariate comparisons and multiple regression, both. Statistical analysis was performed using PASW Statistics 18.

Ethical approval for this research was granted by regional ethical committee for biomedical research in 2010-12-08 number 158200-12-268-61.

## 3. Results

### 3.1. Clinical Data

Both patients and controls were of a similar age, 52.6 years in average and 52 in median, and 87.2% were female. Patients who were entering the study had increased level of triglycerides and decreased HDL-C and tended to have less body mass index as compared to their healthy counterparts. Patients and controls did not meet the criteria of dyslipidaemia; cardiovascular events and diabetes were also exclusion criteria for this study (Table 1). The disease duration of SSc patients varied from 1 to 40 years. The majority of them (74.5%) were of limited subtype of SSc. They all underwent capillaroscopy evaluation with a result showing almost half of the patients being in late stage of the disease. Calcium channel blockers were the standard treatment for 78.7% of patients (Table 2). Up to twenty percent were using either methotrexate or hydroxychloroquine or azathioprine and half of them were taking steroids during the last 6 months before entering the study.

### 3.2. Vascular Measurements Data

We performed a thorough examination of large and medium arteries assessing carotid artery wall and its stiffness and carotid femoral and carotid radial segment of conductive arteries. Arterial wall properties of CCA, and conductive arteries, and wave reflection measurements as well as augmentation index were not different between the study participants (Table 3). The univariate comparison between the groups showed that microvascular component was more pronounced if compared to macrovascular alterations. In particular, the capillary flow during occlusion was comparable between the groups. None of the three laser Doppler measurements taken before and after occlusion showed differences in capillary flow in response to occlusion. However, the reaction to temperature changes, namely, heating and cooling, was different in patients and in healthy controls.

Rest and maximal flow in response to heating were significantly lower among patients, meaning that the small vessels respond poorly to high temperature applications and they take more time to reach the maximal flow. Cold effect was extremely expressed in patients setting as it reduced the capillary flow to minimal possible values. In parallel, RHI reflecting endothelium-dependent postocclusive reaction of small vessels in fingers was lower in systemic SSc patients and considered abnormal (1.50 (0.53) and 2.00 (0.52), *P* < 0.001).

### 3.3. Skin Autofluorescence Data and Predictors of AGE Score

The accumulation of AGE products measured by skin AF was more prominent in SSc patients than in healthy patients, 2.23 (0.54) and 1.90 (0.52), respectively (*P* = 0.007). Considering AGE measurement as a possible cumulative reflection of oxidative stress and cardiovascular damage in SSc, we took further steps to relate it to the profound vascular measurements performed to our study patients.

To examine which of large and small vessels characteristics can explain changes in skin AF and whether it could be related to the disease itself, we have built the stepwise multiple regression model.

The following variables, age of patient or healthy person, SSc as a disease coded (present, 1; absent, 0), FMD (%), cfPWV, crPWV, standardized AIx, IMS/CCA diameter ratio, RHI, capillary rest flow and percentage change during occlusion, and minimal flow when cooling and maximal flow when warming, were entered into the model as independent variables and possible predictors of dependent variable, namely, AGE score.

The final regression equation in step four predicting the AGE score included crPWV (not cfPWV), IMS/CCA diameter ratio, capillary flow percentage change during occlusion, and systemic sclerosis as a disease* per se*. The coefficient of determination of a final model (adjusted *R* ^2^) was 0.347, indicating that these variables explain almost 35 percent of the variance in the total AGE score (Table 4). All the four independent variables had the same direction effect on AGE score. AGE score increased, meaning greater accumulation of advanced glycation end products when crPWV, IMT/CCA diameter ratio, and capillary flow percentage change increased, and it was linked to systemic sclerosis* per se*.

## 4. Discussion

In recent decades, many studies have shown an increase in AGE products' accumulation stimulated by a normal process of ageing, pathological alterations during diabetes [12–14], and renal diseases [15, 16]. Increase in AGE products is also considered as a biomarker for severe complications in cardiovascular disease patients [17–20]. The formation of AGE products is a result of the nonenzymatic reaction between sugars and free amino groups of proteins [21]. According to the description by Nienhuis et al., the oxidative stress which favours the formation of AGE products is able to modulate cellular functions by activation of receptor for advanced glycation end products (RAGE) [22]. As RAGE expression is increased in an inflammatory environment that is present in patients with systemic autoimmune diseases, these patients are especially prone for the deleterious effects of AGE product. Interaction between AGE and RAGE leads to activation of intracellular signalling and subsequent expression of adhesion molecules, chemokines, and proinflammatory cytokines, as well as upregulation of RAGE itself. The AGE-RAGE interaction might act as a proinflammatory loop in these patients, contributing to chronic low grade inflammation, which makes these individuals more prone to development of accelerated atherosclerosis. The soluble form of RAGE (sRAGE), however, is able to act as a decoy to avoid interaction of RAGE with its proinflammatory ligands (AGEs, HMGB1, and S100 proteins). sRAGE concentration was found to be decreased during chronic inflammation. The use of measuring circulating sRAGEs may prove to be a valuable vascular biomarker which may represent a future therapeutic target in chronic inflammatory diseases [21]. The research on AGE-RAGE interaction can provide new ways for blocking the RAGE-induced signalling pathways and, therefore, has potential therapeutic application [23].

We conducted a systematic review of English language citation in PUBMED database referring to the keywords* rheumatic disease* OR* autoimmune disease* OR* systemic sclerosis* AND* skin autofluorescence* OR* advanced glycation end products*. We found twenty relevant publications, and eight of them were related to SSc. Despite substantial knowledge about AGE products' role in inflammatory disorders, its relation to SSc remains scarce and fragmented. The role of AGE in systemic autoimmune diseases is well described for systemic lupus erythematosus [24–26] and to a lesser degree for rheumatoid arthritis [27, 28], however, and only a few publications refer directly to systemic sclerosis [6, 7]. The first attempt to describe it was taken by Yoshizaki et al. [29]. Their study showed that the serum levels of HMGB-1 and sRAGE levels were higher in SSc patients compared to controls and had more frequent involvement of several organs, higher Rodnan total skin thickness score, and immunological abnormalities compared to those with normal levels. Similarly, HMGB-1 and sRAGE levels in animal SSc models were higher than those in control mice. In a later study, Davies et al. [30] demonstrated that SSc patients with skin calcinosis show increased expression of AGE products and RAGE compared to controls; limited SSc and diffuse SSc patients, however, show no consistent evidence of relation to autoantibody status, clinical or histological skin score, or patient age.

An indirect and noninvasive investigation of accumulation of AGE products in SSc patients using an AGE Reader on skin was further undertaken by two groups, which resulted in different conclusions. Hettema et al. in 2011 demonstrated that skin autofluorescence, a marker of tissue AGE accumulation, is not increased in SSc patients [6]. Skin autofluorescence was assessed by measuring UV-A light excitation-emission matrices in 41 SSc patients and 41 age- and sex-matched controls. In this study, an adapted setup of the AGE Reader was used. Namely, they used the Excitation-Emission Matrix Scanner (EEMS), which allows to determine skin autofluorescence (AF-EEMS) and to discriminate between autofluorescence spectra from different fluorophores. When analysing SSc patients and controls together, in multivariate analysis, only age and use of agents intervening in the renin-angiotensin system were independently associated with AF-EEMS. The same device manufactured by the same group in Groningen for AGE product accumulation was used by our group as well. In contrast to that study, Murray et al. in 2012, however, got to different conclusions, when investigated skin autofluorescence in 16 patients with diffuse cutaneous SSc, 15 patients with limited cutaneous SSc, 15 patients with primary Reynauld phenomena, and 13 patients with morphea. Autofluorescence was significantly increased in patients with diffuse cutaneous SSc but not limited cutaneous SSc and not in controls [7]. While examining the group of different subtypes of SSc patients, we demonstrated that AGE accumulation is dependent on a number of vascular factors and the disease itself.

The conflicting results of these two studies drew an immediate attention from both groups and Hettema and Smit responded with remark that different measurement systems and different locations were tested [31] while Murray et al. considered the measurement system less important [32]. He emphasized the importance of different subtypes of a disease because increased skin fluorescence was found predominating in patients with diffuse cutaneous SSc. Of note, among our patients, almost 26 percent from all were with diffuse subtype of disease.

The role of oxidative stress compounds in SSc is still poorly explained and the real value of cross-sectional investigation is not truly understood. Future studies in prospective way may add to our understanding of how and when the biochemical alterations translate into clinically meaningful outcomes during the development of accelerated atherosclerosis.

## 5. Conclusions

Greater accumulation of AGE products in SSc patients as compared to healthy controls was observed in our study. We conclude that AGE product accumulation measured by skin autofluorescence depends on SSc itself. AGE product accumulation is also associated with carotid radial pulse wave velocity, intima media/carotid artery diameter ratio, and capillary flow percentage change during occlusion. This suggests that in future AGE may play a role in predicting early atherosclerosis and cardiovascular events; however, follow up studies will be required to confirm our findings.

## Figures and Tables

**Table 1:** Main characteristics of 47 systemic sclerosis patients matched by age and gender to 47 healthy controls.

	Systemic sclerosis patients *n* = 47	Healthy controls *n* = 47	*P*
Mean age, years Median	52.64 (SD 11.20) 52	52.57 (SD 7.69) 52	0.974
Gender, female, nr (%)	41 (87.2%)	41 (87.2%)	1.00
Body mass index, kg/m^2^	24.27 (SD 4.63)	26.09 (SD 4.50)	0.06
Total cholesterol, mmol/L	5.80 (1.46)	6.22 (1.13)	0.132
Low density lipoprotein cholesterol, mmol/L	3.6651 (SD 1.33)	4.1088 (SD 0.99)	0.083
High density lipoprotein cholesterol, mmol/L	1.35 (SD 0.38)	1.57 (SD 0.34)	0.006
Triglycerides, mmol/L	1.53 (SD 0.68)	1.15 (SD 0.53)	0.005

**Table 2:** Clinical characteristics of 47 systemic sclerosis patients.

Disease duration, years	12.70 (SD 9.52), min–max (1–40)
Rodnan skin thickness score	13.30 (SD 7.21), min–max (2–30)
Subtype of systemic sclerosis	
Limited nr, %	35, 74.5%
Diffuse nr, %	12, 25.5%
Capillaroscopy evaluation	
Active nr, %	17, 36.2%
Late nr, %	20, 42.6%
Early nr, %	10, 21.3%
Treatment during the last half a year	
ACE inhibitors nr, %	8, 17.0%
Calcium channels blockers nr, %	37, 78.7%
Methotrexate nr, %	8, 17.0%
Azathioprine nr, %	8, 17.0%
Antimalarials nr, %	5, 10.64%
Steroids nr, %	23, 48.94%

**Table 3:** Arterial wall or capillary flow assessment and comparison between systemic sclerosis and healthy controls.

Measurement	Systemic sclerosis patients (*n* = 47)	Healthy controls (*n* = 47)	*P*
*Carotid artery wall assessment*			
(IMT/CCA) *∗* 100 mean of right and left CCA	9.34 (3.81–12.99)	9.56 (6.90–49.53)	0.141
Stiffness of carotid arteries (mean of right and left)	3.80 (SD 1.67)	3.75 (SD 1.47)	0.882
*Arterial stiffness and wave reflection measurements*			
Mean blood pressure, mmHg	93.98 (SD 12.81)	96.60 (SD 7.93)	0.237
Pulse wave velocity (carotid femoral), m/sec	7.53 (SD 1.70)	7.51 (SD 1.30)	0.962
Pulse wave velocity (carotid radial), m/sec	8.96 (SD 1.60)	8.61 (SD 1.19)	0.227
Augmentation index (adj. for 75 heart rate)	26.02 (SD 12.08)	24.89 (SD 9.66)	0.618
*Laser Doppler measurements of capillary flow*			
Postocclusive hyperaemia			
Rest flow median before occlusion, PU (min–max)	29.51 (8.00–225.74)	32.66 (8.5–171.99)	0.308
Time to peak hyperaemia, sec	00:34.410 (00:13.860–04:55.930)	00:33.755 (00:05.860–05:37.710)	0.121
Percentage change median between rest and peak flow (min–max)	346.71 (7.10–2.344.74)	383.46 (0.83–909.67)	0.546
Thermal response			
Rest flow median before heating, PU, (min–max)	27.71 (0.81–114.02)	46.53 (4.66–388.74)	0.008
Maximal flow in response to heating, PU	217.01 (SD 150.05)	354.95 (SD 144.41)	<0.001
Time to maximal flow	00:23:47 (00:17:59–00:28:35)	00:22:31 (00:16:31–00:28:01)	0.097
Cold stimulation			
Rest flow median before cooling, PU, (min–max)	84.94 (4.49–388.18)	165.10 (10.67–635.23)	0.178
Minimal flow in response to cooling, PU, (min–max)	11.00 (−0.10–196.10)	72.40 (2.10–435.90)	0.002
Time to minimal flow	00:07:09 (00:05:52–00:09:44)	00:06:29 (00:05:04–00:09:58)	0.052
*Ultrasound assessment of endothelial function in brachial artery*			
Flow mediated dilatation, percentage change	3.24 (SD 2.19)	3.83 (SD 2.48)	0.227
*Peripheral arterial tonometry*			
Reactive hyperaemia index	1.50 (SD 0.53)	2.00 (SD 0.52)	<0.001
*Skin autofluorescence*			
Autofluorescence index	2.23 (SD 0.54)	1.90 (SD 0.47)	0.007

**Table 4:** Predictors of an advanced glycation end product score.

Advanced glycation end product score	Independent variables	Standardized coefficient beta	Statistical significance for standardized coefficient beta

Adjusted determination coefficient for stepwise multiple regression model *R* ^2^ = 0.347 *P* < 0.001 Constant 0.642	Pulse wave velocity (carotid radial), m/sec	0.297	0.010
	(IMT/CCA) *∗* 100	0.388	0.002
	Percentage change median between rest and peak flow	0.245	0.032
	Systemic sclerosis (1)/healthy (0)	0.323	0.007

## References

[1] Sitia S., Atzeni F., Sarzi-Puttini P. (2009). Cardiovascular involvement in systemic autoimmune diseases. *Autoimmunity Reviews*.

[2] Soltész P., Kerekes G., Dér H. (2011). Comparative assessment of vascular function in autoimmune rheumatic diseases: considerations of prevention and treatment. *Autoimmunity Reviews*.

[3] Zinger H., Sherer Y., Shoenfeld Y. (2009). Atherosclerosis in autoimmune rheumatic diseases—mechanisms and clinical findings. *Clinical Reviews in Allergy & Immunology*.

[4] Hahn B. H., Grossman J., Chen W., McMahon M. (2007). The pathogenesis of atherosclerosis in autoimmune rheumatic diseases: roles of inflammation and dyslipidemia. *Journal of Autoimmunity*.

[5] Noordzij M. J. (2013). Skin autofluorecence in atherosclerotic disease. *Skin Autofluorescence and Atherosclerosis*.

[6] Hettema M. E., Bootsma H., Graaff R., De Vries R., Kallenberg C. G. M., Smit A. J. (2011). Skin autofluorescence, as marker of accumulation of advanced glycation endproducts and of cumulative metabolic stress, is not increased in patients with systemic sclerosis. *International Journal of Rheumatology*.

[7] Murray A. K., Moore T. L., Manning J. B., Griffiths C. E. M., Herrick A. L. (2012). Noninvasive measurement of skin autofluorescence is increased in patients with systemic sclerosis: an indicator of increased advanced glycation endproducts?. *Journal of Rheumatology*.

[8] Van Bortel L. M., Laurent S., Boutouyrie P. (2012). Expert consensus document on the measurement of aortic stiffness in daily practice using carotid-femoral pulse wave velocity. *Journal of Hypertension*.

[9] Boignard A., Salvat-Melis M., Carpentier P. H. (2005). Local hyperemia to heating is impaired in secondary Raynaud's phenomenon. *Arthritis Research & Therapy*.

[10] Kuvin J. T., Patel A. R., Sliney K. A. (2003). Assessment of peripheral vascular endothelial function with finger arterial pulse wave amplitude. *American Heart Journal*.

[11] Meerwaldt R., Graaf R., Oomen P. H. N. (2004). Simple non-invasive assessment of advanced glycation endproduct accumulation. *Diabetologia*.

[12] Barlovic D. P., Soro-Paavonen A., Jandeleit-Dahm K. A. M. (2011). RAGE biology, atherosclerosis and diabetes. *Clinical Science*.

[13] Piarulli F., Sartore G., Lapolla A. (2013). Glyco-oxidation and cardiovascular complications in type 2 diabetes: a clinical update. *Acta Diabetologica*.

[14] Vistoli G., De Maddis D., Cipak A., Zarkovic N., Carini M., Aldini G. (2013). Advanced glycoxidation and lipoxidation end products (AGEs and ALEs): an overview of their mechanisms of formation. *Free Radical Research*.

[15] Busch M., Franke S., Rüster C., Wolf G. (2010). Advanced glycation end-products and the kidney. *European Journal of Clinical Investigation*.

[16] Nishizawa Y., Koyama H., Inaba M. (2012). AGEs and cardiovascular diseases in patients with end-stage renal diseases. *Journal of Renal Nutrition*.

[17] Del Turco S., Basta G. (2012). An update on advanced glycation endproducts and atherosclerosis. *BioFactors*.

[18] Prasad A., Bekker P., Tsimikas S. (2012). Advanced glycation end products and diabetic cardiovascular disease. *Cardiology in Review*.

[19] Sell D. R., Monnier V. M. (2012). Molecular basis of arterial stiffening: role of glycation—a mini-review. *Gerontology*.

[20] Giacco F., Brownlee M. (2010). Oxidative stress and diabetic complications. *Circulation Research*.

[21] Maillard-Lefebvre H., Boulanger E., Daroux M., Gaxatte C., Hudson B. I., Lambert M. (2009). Soluble receptor for advanced glycation end products: a new biomarker in diagnosis and prognosis of chronic inflammatory diseases. *Rheumatology*.

[22] Nienhuis H. L. A., Westra J., Smit A. J., Limburg P. C., Kallenberg C. G. M., Bijl M. (2009). AGE and their receptor RAGE in systemic autoimmune diseases: an inflammation propagating factor contributing to accelerated atherosclerosis. *Autoimmunity*.

[23] Musumeci D., Roviello G. N., Montesarchio D. (2014). An overview on HMGB1 inhibitors as potential therapeutic agents in HMGB1-related pathologies. *Pharmacology and Therapeutics*.

[24] Martens H. A., Nienhuis H. L. A., Gross S. (2012). Receptor for advanced glycation end products (RAGE) polymorphisms are associated with systemic lupus erythematosus and disease severity in lupus nephritis. *Lupus*.

[25] Ma C.-Y., Ma J.-L., Jiao Y.-L. (2012). The plasma level of soluble receptor for advanced glycation end products is decreased in patients with systemic lupus erythematosus. *Scandinavian Journal of Immunology*.

[26] Urbonaviciute V., Voll R. E. (2011). High-mobility group box 1 represents a potential marker of disease activity and novel therapeutic target in systemic lupus erythematosus. *Journal of Internal Medicine*.

[27] Vytášek R., Šedová L., Vilím V. (2010). Increased concentration of two different advanced glycation end-products detected by enzyme immunoassays with new monoclonal antibodies in sera of patients with rheumatoid arthritis. *BMC Musculoskeletal Disorders*.

[28] Ji J. D., Woo J.-H., Choi S. J., Lee Y. H., Song G. G. (2009). Advanced glycation end-products (AGEs): a novel therapeutic target for osteoporosis in patients with rheumatoid arthritis. *Medical Hypotheses*.

[29] Yoshizaki A., Komura K., Iwata Y. (2009). Clinical significance of serum HMGB-1 and sRAGE levels in systemic sclerosis: association with disease severity. *Journal of Clinical Immunology*.

[30] Davies C. A., Herrick A. L., Cordingley L., Freemont A. J., Jeziorska M. (2009). Expression of advanced glycation end products and their receptor in skin from patients with systemic sclerosis with and without calcinosis. *Rheumatology*.

[31] Hettema M., Smit A. J. (2013). Local differences in skin autofluorescence may not reflect similar differences in oxidative stress exposure. *Journal of Rheumatology*.

[32] Murray A. K., Griffiths C. E. M., Herrick A. (2013). Dr. Murray, et al reply. *The Journal of Rheumatology*.

